# Brain herniation (encephalocele) into arachnoid granulations: prevalence and association with pulsatile tinnitus and idiopathic intracranial hypertension

**DOI:** 10.1007/s00234-022-02934-9

**Published:** 2022-03-25

**Authors:** Eric R. Smith, M. Travis Caton, Javier E. Villanueva-Meyer, Justin Remer, Laura B. Eisenmenger, Amanda Baker, Vinil N. Shah, Adelyn Tu-Chan, Karl Meisel, Matthew R. Amans

**Affiliations:** 1 Department of Radiology and Biomedical Imaging, University of California San Francisco, 505 Parnassus Ave., San Francisco, CA 94117 USA; 2 Department of Radiology, University of Wisconsin, Madison, WI USA; 3 Department of Neurology, University of California San Francisco, San Francisco, CA USA

**Keywords:** Brain herniation, Arachnoid granulation, Idiopathic intracranial hypertension, Pulsatile tinnitus, Magnetic resonance imaging

## Abstract

**Purpose:**

Brain herniation into arachnoid granulations (BHAG) of the dural venous sinuses is a recently described finding of uncertain etiology. The purpose of this study was to investigate the prevalence of BHAG in a cohort of patients with pulsatile tinnitus (PT) and to clarify the physiologic and clinical implications of these lesions.

**Methods:**

The imaging and charts of consecutive PT patients were retrospectively reviewed. All patients were examined with MRI including pre- and post-contrast T1- and T2-weighted sequences. Images were reviewed separately by three blinded neuroradiologists to identify the presence of BHAG. Their location, signal intensity, size, presence of arachnoid granulation, and associated dural venous sinus stenosis were documented. Clinical records were further reviewed for idiopathic intracranial hypertension, history of prior lumbar puncture, and opening pressure.

**Results:**

Two hundred sixty-two consecutive PT patients over a 4-year period met inclusion criteria. PT patients with BHAG were significantly more likely to have idiopathic intracranial hypertension than PT patients without BHAG (OR 4.2, CI 1.5–12, *p* = 0.006). Sixteen out of 262 (6%) patients were found to have 18 BHAG. Eleven out of 16 (69%) patients had unilateral temporal or occipital lobe herniations located in the transverse sinus or the transverse-sigmoid junction. Three out of 16 (19%) patients had unilateral cerebellar herniations and 2/16 (13%) patients had bilateral BHAG.

**Conclusion:**

In patients with PT, BHAG is a prevalent MRI finding that is strongly associated with the clinical diagnosis of IIH. The pathogenesis of BHAG remains uncertain, but recognition should prompt comprehensive evaluation for IIH.

## Introduction

Pulsatile tinnitus (PT) is the sensation of a “whooshing” sound that corresponds to one’s heartbeat, which affects nearly 3 million Americans [1–4]. PT can be severely debilitating and has been associated with anxiety, depression, and suicide. While there are numerous vascular and nonvascular conditions associated with PT, idiopathic intracranial hypertension (IIH) is common with significant morbidity if left untreated. There are several classic imaging findings of IIH, the commonest being transverse sinus (TS) stenosis seen in 94% of patients with IIH on a recent study by Morris et al. [5].

Increased scrutiny of these stenoses during imaging workup has led to the increased recognition of associated brain herniations into arachnoid granulations (BHAG) of the dural venous sinuses. A previously underrecognized entity, the pathophysiology of these herniations is unknown. Previous literature has variably referred to this entity as encephaloceles [6] or internal cephaloceles [5]. However, as these herniations occur in stereotypical locations of arachnoid granulations, it is thought that brain herniations occur within these outpouchings, and therefore the term brain herniation into arachnoid granulations (BHAG) is the favored terminology which will be used in this manuscript [7]. BHAG are of unknown clinical significance, and their incidence in the PT population remains unknown.

To further understand this phenomenon, we retrospectively analyzed a cohort of patients seen at our institution’s Pulsatile Tinnitus Clinic to identify the presence of BHAG and explore correlations between the presence of BHAG with clinical diagnosis of IIH.

## Methods

### Patient selection

We retrospectively reviewed imaging and charts of consecutive adult patients seen between January 2015 and May of 2019 in our Pulsatile Tinnitus Clinic using a study protocol approved by our Institutional Review Board. Inclusion criteria included age greater than 18, confirmed clinical diagnosis of PT, and complete T1 and T2 MR imaging with MR venogram.

### Imaging and image analysis

MR imaging was performed on 1.5 and 3.0 T scanners and all examinations contained angiographic sequences (MR venogram) in addition to standard morphologic sequences. In most cases, post-contrast 3D SPGR (spoiled gradient recalled acquisition in steady state) was available for evaluation. Although MR imaging was a requisite for inclusion, all available cross-sectional imaging of the brain was analyzed in each patient. Examinations were reviewed separately by three experienced neuroradiologists blinded to patient’s clinical data (JEV-M, LBE, VNS). Any discrepancies were resolved by consensus with a fourth rater, an experienced diagnostic and interventional neuroradiologist (MRA).

In each case, raters assessed (1) the presence of BHAG, (2) the parenchymal origin of BHAG and continuity of the lesion with adjacent gyral tissue, (3) the location of herniation into dural venous sinus, (4) degree of sinus stenosis (> 50%), and (5) the presence or absence of signal abnormality of the BHAG. For cases with multiple brain herniations, each lesion was analyzed separately.

In addition, raters evaluated each case for the presence or absence of conventional MR imaging signs of IIH including empty sella, enlarged Meckel’s caves, optic nerve buckling and sheath subarachnoid space prominence, flattening of the posterior globe and optic disc protrusion, narrowing of the dural venous sinuses, and thinning of the calvarium and skull base. Laterality of the dominant dural venous sinus pathway and any other possible causes of PT identified on the imaging were also recorded (Table 1).

**Table 1:** Patient and imaging characteristics

Case	Age at diagnosis	Sex	PT laterality	IIH on imaging	Involved brain	Involved sinus	Sinus Dominance	Arachnoid granulation	Sinus stenosis	Other findings	Parenchymal change
1	44	F	Right	Present	Left cerebellar	Transverse	Right	Bilateral	Bilateral	R osseous thinning	Absent
2	44	F	Right	Present	Left posterior temporal	Transverse/sigmoid	Right	Bilateral	Bilateral	R osseous thinning	Absent
2	44	F	Right	Present	Right posterior temporal	Transverse/sigmoid	Right	Bilateral	Bilateral	R osseous thinning	Absent
3	31	F	Right	Present	Right posterior temporal	Transverse/sigmoid	Right	Bilateral	Bilateral	No	Absent
4	51	F	Right	Present	Left occipital	Transverse	Right	Left	Left	R osseous thinning	Absent
5	36	F	Right	Present	Right posterior temporal	Transverse/sigmoid	Right	Right	Right	R osseous thinning	Absent
6	69	M	Right	Absent	Right posterior temporal	Transverse/sigmoid	Right	Right	None	R dolichoectasia	Absent
7	25	F	Right	Absent	Right occipital	Transverse	Codominant	Right	None	No	Present
8	60	F	Right	Absent	Left occipital	Transverse	Codominant	Left	Left	No	Present
9	63	M	Left	Absent	Left occipital	Transverse	Codominant	Left	None	No	Present
10	42	M	Right	Present	Left posterior temporal	Transverse/sigmoid	Right	Bilateral	Bilateral	No	Absent
11	79	M	Right	Absent	Left occipital	Transverse	Right	Bilateral	None	R high riding jugular bulb	Present
11	79	M	Right	Absent	Right cerebellar	Transverse	Right	Bilateral	None	R high riding jugular bulb	Present
12	44	F	Left	Absent	Left posterior temporal	Transverse/sigmoid	Right	Left	Left	No	Absent
13	41	F	Right	Present	Left cerebellar	Transverse	Right	Bilateral	Bilateral	No	Present
14	28	F	Right	Present	Right occipital	Transverse	Right	Right	Right	No	Present
15	71	F	Left	Absent	Right cerebellar	Transverse	Left	Bilateral	Bilateral	No	Absent
16	26	F	Right	Present	Left posterior temporal	Transverse/sigmoid	Codominant	Left	Left	No	Absent

### Clinical data review

Medical records of all patients from this cohort were reviewed for age, sex, BMI, duration of PT, documented clinical diagnosis of IIH using the modified Dandy Criteria [8–11], history of prior lumbar puncture, and opening pressure.

### Statistical analysis

Patients from our PT cohort were divided amongst those with MR imaging findings of BHAG and those without BHAG. Within these two groups, further subdivision was performed between patients with and without clinical diagnosis of IIH and comparison was made to identify the significance of BHAG in increasing the odds of IIH, specifically for patients with PT. Statistical analysis was then performed using chi square and odds ratio tests to assess for intergroup differences. Significance was defined as *p* < 0.05.

## Results

Review of our Pulsatile Tinnitus Clinic database identified 262 patients over the 4-year inclusion period. The overall prevalence of BHAG in this population was 6% (16/262 patients, 18 total BHAG lesions). Median age was 44 years (range 25–79). 81% were females. Median time to presentation from symptom onset was 12 months (range 1–70 months).

Of the 16 patients with BHAG, 11 (69%) had unilateral temporal or occipital herniations into the transverse sinus or the transverse-sigmoid junction, respectively. Three patients (19%) had unilateral cerebellar BHAG. One patient (6%) had bilateral temporal BHAG into the transverse-sigmoid junctions, and another patient had left occipital and contralateral right cerebellar BHAG into the transverse sinuses. Of patients with unilateral BHAG, 7/14 (50%) occurred on the same side as PT symptoms. The remaining 7/14 (50%) patients had unilateral BHAG *contralateral* to side of symptomatic PT.

Abnormal T2-hyperintense signal of the herniated brain parenchyma was seen in 7/18 (39%) BHAG. No other MR signal abnormality was noted, including enhancement or reduced diffusion.

Twelve out of 16 (75%) patients with BHAG had a unilateral dominant dural venous sinus. The dominant venous sinus pathway was ipsilateral to PT symptoms in 11/12 (92%) patients. Eight out of 16 (50%) patients had associated ipsilateral osseous thinning at the dominant transverse-sigmoid sinus junction, dolichoectasia, or high riding jugular bulbs. Patient and imaging characteristics are summarized in Table 1.

**Fig. 1:**
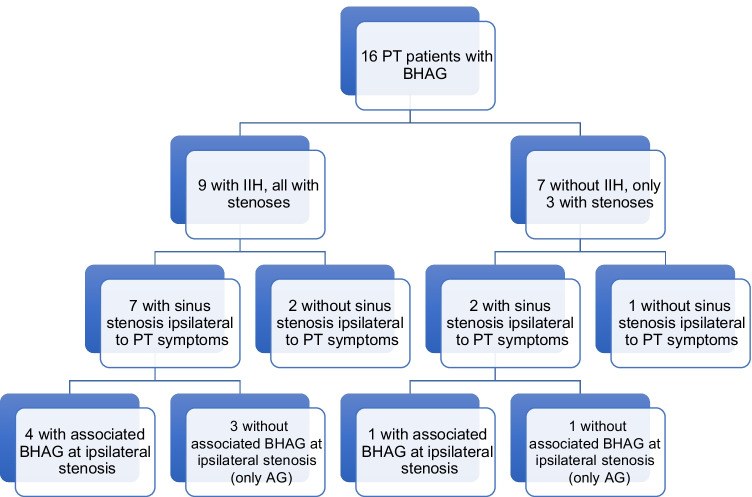
Patient flowchart depicting dural venous sinus stenosis laterality and frequency of associated BHAG in IIH and non-IIH sub-cohorts

In PT patients with BHAG, 9/16 (56%) had clinical diagnosis of IIH as determined by modified Dandy Criteria with 7/9 (78%) confirmed by lumbar puncture with a median opening pressure of 28 cm of water. All 9 IIH patients had focal stenoses in the dural venous sinuses. Of these patients with BHAG and IIH, 7/9 (78%) had sinus stenosis ipsilateral to PT symptoms (Fig. 1). Patient clinical findings, treatments, and outcomes for this group are depicted in Table 2. In 246 PT patients without BHAG, 55/246 (23%) had a clinical diagnosis of IIH per the modified Dandy Criteria, with 39/55 (71%) confirmed by lumbar puncture with a median opening pressure of 27 cm of water.

**Table 2:** Patient clinical findings, treatment, and outcomes

Case	Age at diagnosis	Sex	BMI	Pulsatile tinnitus laterality	Duration (months)	OP (cm H20)	Clinical Diagnosis IIH	Treatment	Follow-up
1	44	F	34	Right	36	36	Yes	Weight loss and Diamox	Resolved
2	44	F	31	Right	70	28	Yes	Right TS stenting	Resolved
3	31	F	40	Right	12	40	Yes	Diamox	Improved
4	51	F	40	Right	24	28	Yes	Diamox	Improved
5	36	F	44	Right	12	Unknown	Yes	Weight loss, low salt	Unknown
6	69	M	40	Right	10	Unknown	No	Dietary changes, cyclobenzaprine	Improved
7	25	F	18	Right	2	Unknown	No	Unknown	Unknown
8	60	F	26	Right	4	Unknown	No	DAVF embolized	Resolved
9	63	M	27	Left	1	Unknown	No	AVF resected	Resolved
10	42	M	49	Right	24	28	Yes	Right TS stenting	Resolved
11	79	M	22	Right	1	Unknown	No	None	Resolved
12	44	F	22	Left	4	12	No	None	Unchanged
13	41	F	40	Right	3	26	Yes	Diamox	Unchanged
14	28	F	36	Right	12	Unknown	Yes	Unknown	Unknown
15	71	F	23	Left	18	Unknown	No	None	Unchanged
16	26	F	51	Right	36	29	Yes	Diamox	Improved

The overall frequency of IIH by modified Dandy Criteria was significantly higher in patients with BHAG than in those without BHAG (OR 4.2, CI 1.5–12, *p* = 0.006).

## Discussion

This study describes the prevalence and clinical and imaging characteristics of BHAG in a large, consecutive cohort of patients undergoing diagnostic evaluation for PT. We identify two key observations in this analysis: First, BHAG are a relatively common imaging finding in PT patients, with a prevalence of 6% of this cohort. This suggests that BHAG may be underreported and/or underrecognized by neuroradiologists. Second, patients with PT and BHAG are significantly more likely to have underlying IIH than PT patients without BHAG (OR 4.2, CI 1.5–12, *p* = 0.006). The latter observation confirms the clinical importance of diagnosing BHAG, which should prompt evaluation for underlying IIH in this patient population. Radiologists, neurologists, otorhinolaryngologists, and other clinicians who care for patients with PT should be aware of this relationship to prevent blindness and other morbidity associated with untreated IIH.

**Fig. 2:**
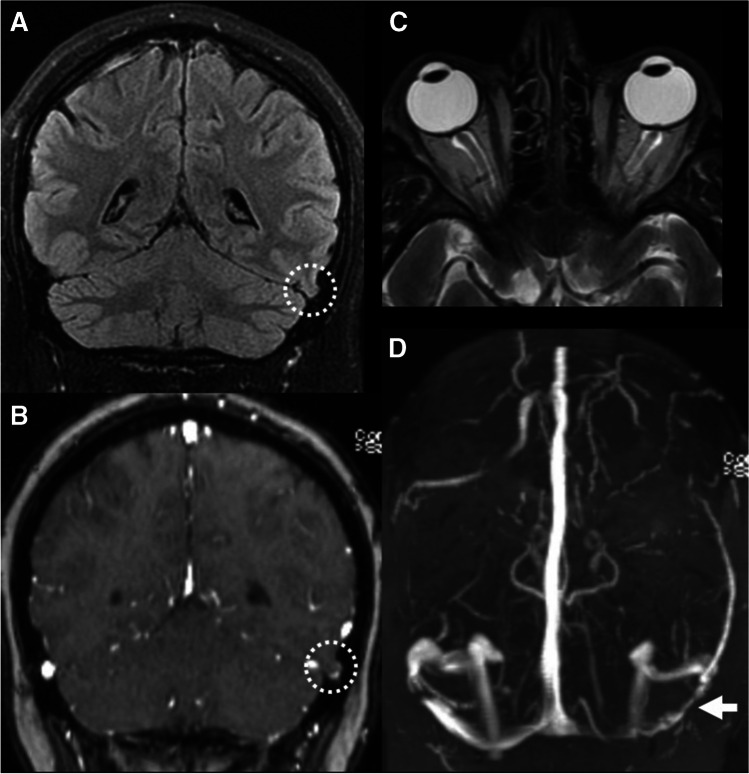
MR imaging example of IIH in the setting of a left transverse BHAG (patient 10 with right-sided PT). **A**, **B** Coronal T2 FLAIR and T1 post-contrast MR images show a small BHAG of the left posterior temporal lobe into the transverse sinus (dashed circle). **C** Axial T2 MR image shows flattening of the posterior globes and protrusion of the optic nerve heads as well as prominent optic nerve sheath subarachnoid space, findings suggestive of elevated intracranial pressure. **D** MR venogram shows bilateral transverse sinus stenoses, most pronounced on the left side at site of the BHAG (white arrow)

The present data suggest that BHAG are likely an underrecognized entity in neuroimaging. This observed prevalence of BHAG in PT patients was substantially higher than a prior estimated prevalence of < 1% in the general population in a study of over 6000 MRI studies [12]. Although BHAG pathogenesis remains obscure, several theories exist to explain their cause. Battal et al. recently described several potential etiologies including: (1) weakness in arachnoid granulations due to protruding veins; (2) transient increase in intracranial pressure; or (3) spontaneous herniation [12]. Previous work has suggested an association between BHAG and increased intracranial pressure, but the association of BHAG in PT patients has not been reported [12]. Our study also builds on prior investigations by showing, for the first time, a strong association between MR findings of BHAG and IIH (Fig. 2), which implies a pathophysiologic relationship between these entities as has previously been hypothesized [13].

Although laterality of BHAG in our cohort did not co-localize with PT symptom side, we did observe a strong relationship between laterality of dural venous sinus dominance and PT symptoms, consistent with prior work [1]. It seems that BHAG can form in enlarged arachnoid granulations in either the dominant or non-dominant venous sinuses, and as such the presence of them does not indicate laterality of symptoms as much as help direct one to the underlying etiology of IIH. Since BHAG location does not match symptom laterality, they are also unlikely to be causing sound in these PT patients. It is more likely that the enlarged arachnoid granulations in the dominant venous sinus are causing PT, and the underlying reason for the enlarged arachnoid granulations is IIH (commonly described as an intrinsic stenosis).

**Fig. 3:**
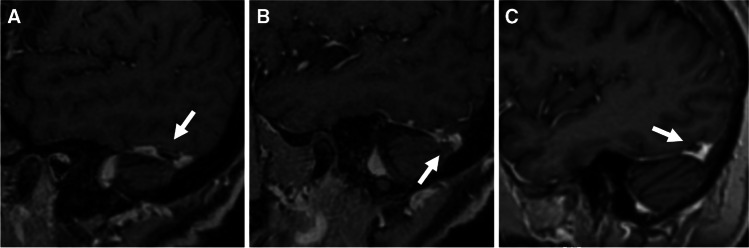
MR imaging example of BHAG compared to “simple” arachnoid granulation. **A** Sagittal T1 post-contrast MR images of patient 11 (right-sided PT) show left occipital BHAG into the transverse sinus, as well as **B** right cerebellar BHAG into the transverse sinus in the absence of IIH. **C** Sagittal T1 post-contrast MR image of a separate patient demonstrates an arachnoid granulation in a similar location without brain herniation

BHAG occurred in typical locations of arachnoid granulations, predominantly in the transverse, sigmoid, and superior sagittal sinuses (Fig. 3). While the majority of BHAG demonstrate MRI signal characteristics equivalent to that of normal brain parenchyma, a minority have abnormal T2/FLAIR hyperintense signal of undetermined etiology. In the current study, 39% of BHAG had associated abnormal parenchymal signal, which is comparable to a prior reported rate of 33% [7]. No other abnormal signal characteristics were found, although abnormal enhancement and reduced diffusion have been reported. It is incumbent on the radiologist to recognize and accurately describe BHAG because these lesions have been documented as seizure foci which may require urgent intervention [14].

Various nomenclature currently exists to describe brain herniations into dural venous sinuses including internal cephaloceles, encephaloceles, or simply “brain herniations” [5–7, 12, 15, 16]. The classic definition of an encephalocele is a brain herniation containing meninges that extends through a defect in the skull base or calvarium. In contrast, brain herniations into arachnoid granulations may occur at the dural venous sinuses without true osseous dehiscence. All instances in our cohort were associated with arachnoid granulations. For these reasons, we advocate the term brain herniations into arachnoid granulations or “BHAG.” Nonetheless, there is question whether BHAG and encephaloceles are interrelated [17]. While the fine analysis is outside the scope of this study, previous pathology literature by Beneke and Wolbach suggests similar CSF pulsation forces and pulling of the arachnoid and pia mater, raising the possibility of overlap in causation [16]. It is unknown whether this is purely coincidental or in fact these are one and the same.

In the setting of PT, BHAG is important to distinguish from other etiologies that result in a stenosis or focal defect of the dural venous sinuses to appropriately guide patient management and treatment. Differential considerations for BHAG include simple arachnoid granulation, thrombus, tumor, and vascular anomaly such as dural arteriovenous fistula [12]. It is important to note that to our knowledge, BHAG have *never* been reported into a thrombosed sinus. As such, identifying a BHAG can help reassure thrombus is not present in that section of vein and the stenotic lesion is an arachnoid granulation, thus obviating the need for anticoagulation in these patients.

Understanding the relationship of BHAG with IIH in patients with PT is of the upmost clinical interest as IIH is a serious and debilitating condition associated with PT. Specifically, the role of imaging to identify venous anatomy variants in patients with both PT and IIH can hold significant value in diagnosing these conditions. Prior work has demonstrated that venous sinus stenosis is the most sensitive MR imaging finding in patients with IIH, occurring in 94% [5]. This reported sensitivity is higher than previous work, possibly due to continued improvements in MRI technique, which will lead to increased recognition and characterization of BHAG. While not all patients with IIH will present with PT, prior studies have demonstrated that sigmoid sinus wall dehiscence can be correlated with PT in IIH patients [18]. Our results build upon these prior studies and suggest that the presence of BHAG may increase the specificity of diagnosing IIH. Future work will continue to clarify the relationship between IIH and BHAG in the PT population.

Pathologic flow patterns in the dural venous sinuses play an important role in the pathogenesis of venous PT [19]. Prior studies have implicated hemodynamics as the causal mechanism of PT in conditions including venous stenoses, sigmoid diverticula, and high riding jugular bulb using computational fluid dynamic, 4D MR velocimetry, and patient-specific 3D printed models [19–25]. Future studies employing these techniques may clarify the venous flow dynamics in PT patients with BHAG and further quantify the role that BHAG play in altering intracranial venous drainage.

Arachnoid granulations *without* associated BHAG have also been associated with IIH and have been implicated as a compensatory mechanism [26]. In these cases, treatment of the underlying IIH with weight loss and medical therapy followed by CSF diversion is most appropriate [27, 28], although venous sinus stenting (VSS) has successfully been used to treat stenotic arachnoid granulations [29].

VSS across an encephalocele has been shown to be safe and feasible [29]. Our current results corroborate this treatment efficacy, with patients demonstrating improvement or resolution of IIH and PT symptoms. Furthermore, a significant component of these patients improved with medical management alone, highlighting the efficacy of non-invasive treatment despite symptomatic stenoses with BHAG. Given the significant correlation of IIH with BHAG in PT patients, future work should analyze stenting of sinuses with BHAG compared to medical management of IIH and its efficacy in resolving PT.

Several limitations of this study merit additional discussion. First, as a retrospective study, from a single quaternary-referral center, these results may not extend to the general population. Although our institution has significant experience with these lesions, we did not investigate the diagnostic test characteristics for BHAG detection on MRI nor did we evaluate inter-radiologist agreement. Given the often-subtle appearance of BHAG, these questions will be important to address in future work. Finally, this retrospective study design is potentially affected by confirmation bias as the study neuroradiologist evaluated the presence of BHAG and neuroimaging features of IIH during a single imaging review. However, the final determination of IIH was independently adjudicated using clinical criteria.

## Conclusion

BHAG are an underrecognized entity and are prevalent in patients with PT. The presence of BHAG is strongly associated with IIH, therefore, these lesions are an important neuroimaging finding which should prompt expedited clinical workup. Future work is needed to clarify the mechanistic relationship underlying this association.

## Data Availability

Data is available upon reasonable request.

## Declarations

The authors declare that they had full access to all the data in this study and the authors take complete responsibility for the integrity of the data and the accuracy of the data analysis.

## References

[1] Krishnan A, Mattox DE, Fountain AJ, Hudgins PA (2006). CT arteriography and venography in pulsatile tinnitus: preliminary results. AJNR Am J Neuroradiol.

[2] Liyanage SH, Singh A, Savundra P, Kalan A (2006). Pulsatile tinnitus. J Laryngol Otol.

[3] Madani G, Connor SEJ (2009). Imaging in pulsatile tinnitus. Clin Radiol.

[4] Harvey RS, Hertzano R, Kelman SE, Eisenman DJ (2014). Pulse-synchronous tinnitus and sigmoid sinus wall anomalies: descriptive epidemiology and the idiopathic intracranial hypertension patient population. Otol Neurotol.

[5] Morris PP, Black DF, Port J, Campeau N (2017). Transverse sinus stenosis is the most sensitive MR imaging correlate of idiopathic intracranial hypertension. Am J Neuroradiol.

[6] Valci L, Dalolio M, Kuhlen D (2017). Intradiploic encephalocele of the primary motor cortex in an adult patient: electrophysiological implications during surgery. J Neurosurg.

[7] Liebo GB, Lane J (Jack) I, Gompel JJV et al (2016) Brain herniation into arachnoid granulations: clinical and neuroimaging features. J Neuroimaging 26:592–598.10.1111/jon.12366 10.1111/jon.12366 27273503

[8] Smith JL (1985). Whence pseudotumor cerebri?. J Clin Neuroophthalmol.

[9] Dandy WE (1937). Intracranial pressure without brain tumor. Ann Surg.

[10] (2018) Headache Classification Committee of the International Headache Society (IHS) The International Classification of Headache Disorders, 3rd edition. Cephalalgia 38:1–211. 10.1177/0333102417738202 10.1177/0333102417738202 29368949

[11] Mollan SP, Davies B, Silver NC (2018). Idiopathic intracranial hypertension: consensus guidelines on management. J Neurol Neurosurg Psychiatry.

[12] Battal B, Hamcan S, Akgun V (2016). Brain herniations into the dural venous sinus or calvarium: MRI findings, possible causes and clinical significance. Eur Radiol.

[13] Sismanis A, Hughes GB, Abedi E (1985). Otologic symptoms and findings of the Pseudotumor Cerebri Syndrome: a preliminary report. Otolaryngol Head Neck Surg.

[14] Yadav T, Shaikh M, Panda S, Khera P (2020). Temporal encephalocele into transverse sinus in an adult with partial seizures: MRI evaluation of a rare site of brain herniation. Indian J Radiol Imaging.

[15] Chan WC, Lai V, Wong YC, Poon WL (2011). Focal brain herniation into giant arachnoid granulation: a rare occurrence. Eur J Radiol Extra.

[16] Malekzadehlashkariani S, Wanke I, Rüfenacht DA, San Millán D (2016). Brain herniations into arachnoid granulations: about 68 cases in 38 patients and review of the literature. Neuroradiology.

[17] Urbach H, Jamneala G, Mader I (2018). Temporal lobe epilepsy due to meningoencephaloceles into the greater sphenoid wing: a consequence of idiopathic intracranial hypertension?. Neuroradiology.

[18] Zhao P, Jiang C, Lv H (2021). Why does unilateral pulsatile tinnitus occur in patients with idiopathic intracranial hypertension?. Neuroradiology.

[19] Amans MR, Haraldsson H, Kao E (2018). MR venous flow in sigmoid sinus diverticulum. Am J Neuroradiol.

[20] Haraldsson H, Leach JR, Kao EI (2019). Reduced jet velocity in venous flow after csf drainage: assessing hemodynamic causes of pulsatile tinnitus. Am J Neuroradiol.

[21] Valluru K, Parkhill J, Gautam A (2020). Sound measurement in patient-specific 3D printed bench models of venous pulsatile tinnitus. Otol Neurotol.

[22] Kao E, Kefayati S, Amans MR (2017). Flow patterns in the jugular veins of pulsatile tinnitus patients. J Biomech.

[23] Kefayati S, Amans M, Faraji F (2017). The manifestation of vortical and secondary flow in the cerebral venous outflow tract: an in vivo MR velocimetry study. J Biomech.

[24] Alexander MD, Meisel KM, Halbach VV (2017). Enlarged condylar veins as a source of pulsatile tinnitus: angiographic features and confirmation with venous balloon test occlusion. Neurographics.

[25] Four dimensional magnetic resonance velocimetry for complex flow in the jugular vein - Acevedo-Bolton - Quantitative Imaging in Medicine and Surgery. https://qims.amegroups.com/article/view/6461/7977. Accessed 14 Feb 2022 10.3978/j.issn.2223-4292.2015.04.09 PMC4559987 26435930

[26] Watane GV, Patel B, Brown D, Taheri MR (2018). The significance of arachnoid granulation in patients with idiopathic intracranial hypertension. J Comput Assist Tomogr.

[27] Starke RM, Wang T, Ding D (2015). Endovascular treatment of venous sinus stenosis in idiopathic intracranial hypertension: complications, neurological outcomes, and radiographic results. Scientific World Journal.

[28] Wakerley B, Tan M, Ting E (2015). Idiopathic intracranial hypertension. Cephalalgia.

[29] Drocton GT, Copelan A, Eisenmenger L (2021). Venous sinus stenting as a treatment approach in patients with idiopathic intracranial hypertension and encephaloceles. Interv Neuroradiol.

